# Nitric oxide in vascular biology: elegance in complexity

**DOI:** 10.1172/JCI176747

**Published:** 2024-02-15

**Authors:** Joseph Loscalzo

**Affiliations:** Department of Medicine, Brigham and Women’s Hospital, Harvard Medical School, Boston, Massachusetts, USA.

As a graduate student at the University of Pennsylvania many years ago, I read journals and wrote my thesis in the Biochemistry Department’s library, the walls of which were adorned with photomicrographs of crystals of hemoglobin. These elegant images were taken by David Drabkin, who is best known for the development of a colorimetric reagent used to quantify hemoglobin in whole blood. He was, however, also an excellent physical biochemist, who first crystallized human hemoglobin and explored its spectrophotometric properties and those of some of its derivatives (1). Among the many derivatives and adducts of hemoglobin that gained increasing importance over the past 60 years is that of nitrosyl-hemoglobin, which forms when deoxy-hemoglobin is exposed to nitric oxide (NO) (2). This interaction with heme iron is one of many biochemical reactions in which NO is engaged and accounts for a plethora of functional effects in mammals (Figure 1). One example includes the activation of guanylyl cyclase that occurs with the binding of NO to the enzyme’s prosthetic heme group. Little did I realize at the time that an important part of my own research career would focus on this deceptively simple heterodiatomic molecule and its pathobiological actions.

## NO and heme in prebiotic and biotic evolution

NO probably first appeared in the prebiotic phase of geochemical evolution approximately 4.5 to 2.5 billion years ago (Bya) from volcanic action and lightning discharges in the Archean atmosphere. With the advent of the “great oxidation event” approximately 2.4 to 2.2 Bya, higher NO oxidation states evolved and, coupled with photochemical and free radical reactions in a water vapor phase, created nitrogen-based acids that would have adverse effects on early biotic evolution (3). The prebiotic evolution of the highly chemically versatile porphyrins (4) set the stage for the generation of heme species, which likely served initially as a means to trap and thereby detoxify NO, limiting its adverse effects on early microbial evolution.

## Effectors of vascular function

As a cardiology fellow, I became intrigued by the mechanism of action of organic nitrates commonly used to treat angina pectoris and acute coronary syndromes. In addition to cyclic GMP–dependent (cGMP-dependent) vascular smooth muscle relaxation, organic nitrates appeared to inhibit platelet activation, albeit at concentrations that far exceed those achieved clinically. Yet, clinical data showed that nitroglycerin did, indeed, prolong bleeding time. Needleman’s observations on the oxidative inactivation of organic nitrates accounting for tolerance and its prevention by thiol species (5) led us to explore the effects of the thiol *N*-acetyl-l-cysteine (NAC) on the antiplatelet effects of organic nitrates (6). We found that thiol species dramatically enhanced platelet inhibition by organic nitrates and did so through the formation of an S-nitrosothiol adduct of NAC, *S*-nitroso-*N*-acetyl-l-cysteine. Importantly, NO does not directly react with thiol or thiolate functionalities, but only does so as the nitrosonium species NO^+^ or via other nitrosating intermediates, such as acidified nitrite generated in the stomach or dinitrosyl iron complexes (DNICs).

Work by Furchgott demonstrated that the endothelial cell generates a substance that is responsible for muscarinic agonist–dependent smooth muscle relaxation, known initially as endothelium-derived relaxing factor or EDRF (7). By the late 1980s, EDRF was identified as NO by Ignarro (8) and independently by Moncada (9), and its vasorelaxing properties were found by Murad to be a consequence of guanylyl cyclase activation (10) via binding to the enzyme’s prosthetic heme group. For their work, Furchgott, Ignarro, and Murad won the Nobel Prize in Medicine or Physiology in 1998.

The identification of NO as EDRF set off a host of studies that attempted to explore its metabolism and biochemistry, its actions in health and disease, and its potential therapeutic effects. NO is synthesized by members of the NO synthase (NOS) family of oxidoreductases, each of which converts l-arginine to l-citrulline and NO. A key member of this family from the vascular perspective is the endothelial isoform eNOS, also known as NOS3, which is responsible for the highly regulated generation of endothelium-derived NO. The chemistry of NO is complex, owing to its multiple redox states and its differential reactivity toward different ROS. While NO is a free radical, it is far less reactive than other biologically relevant free radical species, allowing it to diffuse over greater distances to bring about its biological actions as it encounters other biochemical coreactants (11).

Apropos of the effects of thiols on nitrovasodilator activity, we first demonstrated that NO can form S-nitrosothiols in vivo with both low-molecular-weight thiols (e.g., glutathione) and proteins. In fact, *S*-nitros(yl)ation reactions are posttranslational modifications of protein thiol functionalities that form a reservoir prolonging the half-life of NO and in some cases altering protein function (12). *S*-Nitrosothiols can undergo trans-*S*-nitrosation reactions to facilitate transfer to specific acceptors (13); a key mechanism for transcellular trans-*S*-nitrosation involves transport into the intracellular environment via catalysis by protein disulfide isomerase (14).

Once endothelium-derived NO has gained access to its effector cell (e.g., the vascular smooth muscle cell or platelet), it binds to guanylyl cyclase’s prosthetic heme group to activate the enzyme, generate cGMP, and lead to cGMP-dependent smooth muscle relaxation or platelet inhibition, respectively. NO also plays a key signaling role downstream of VEGF in promoting angiogenesis (15). In addition to the direct antiplatelet effects of endogenous endothelium–derived NO, platelet-derived NO limits recruitment of platelets to the growing platelet thrombus, constraining platelet-dependent hemostasis (16). NO-generating vasodilators also impair vascular smooth muscle cell proliferation (17), an observation that served as the basis for an NO-therapeutic strategy to limit vascular smooth muscle cell proliferation following vascular injury with de-endothelialization such as with angioplasty (18).

## NO in vascular pathobiology and disease

NO insufficiency as a manifestation of endothelial dysfunction is a key determinant of many vascular pathobiologies. A decrease in bioactive NO caused by hypercholesterolemia-induced endothelial dysfunction was found to impair vasodilation in forearm vessels (19). Complementarily, NO was found to decrease endothelial cell activation and adhesion molecule expression in response to inflammatory cytokines critical for atherogenesis (20). Two experiments of nature also provide evidence for the importance of NO in vascular homeostasis. First, in the rare disorder of lysinuric protein intolerance caused by a mutation in the dibasic amino acid transporter SLC7A7, l-arginine and NO levels are substantially reduced and accompanied by impaired coronary perfusion and a prothrombotic state, abnormalities that were reversed by the administration of exogenous l-arginine (21). Second, two children with a history of arterial thrombosis were found to have a deficiency of bioactive NO secondary to decreased plasma glutathione peroxidase activity (22). Plasma glutathione peroxidase (GPx-3) is a key extracellular antioxidant enzyme that reduces hydrogen and lipid peroxides to water and lipid alcohols, respectively. A deficiency of GPx-3 is associated with increased peroxynitrite formation, impaired NO bioactivity, and enhanced platelet-dependent thrombosis, as shown in a genetic murine model (23). In addition to these rare variants, more common genetic polymorphisms in the eNOS gene *NOS3* and in the guanylyl cyclase isoform 1A3 gene *GUCY1A3* have been shown to convey an increased risk of atherothrombotic disease (24), while gain-of-function variants in *NOS3* appear to be atheroprotective (25).

## Future directions

The history of the vascular biology of NO is rich with links to prebiotic geochemistry, early vascular therapeutics, and complex redox biochemistry. While the field has continued to advance over the past four decades, there remain many opportunities for further developments in redox biology, with a clearer understanding of the predictive determinants of NO’s reactivity toward other free radicals and radical anions and their downstream reactions; in therapeutics, with targeted delivery to specific tissues, or with agents that can enhance endogenous NO production by NO synthases; in genomics, with increased precision in identifying genomic determinants of NO generation or inactivation; in metagenomics, with a clearer understanding of the gut, oral, and dermal microbiome and its role(s) in modulating endogenous NO_x_ pools; and in diagnostics, with expired gas, plasma, or other bodily fluid patterns of NO_x_ species and their association with disease or treatment. The future of vascular NO research will be complex, to be sure, but promises to offer an increasingly detailed view of the biological elegance of this remarkable molecule.

## Figures and Tables

**Figure 1:**
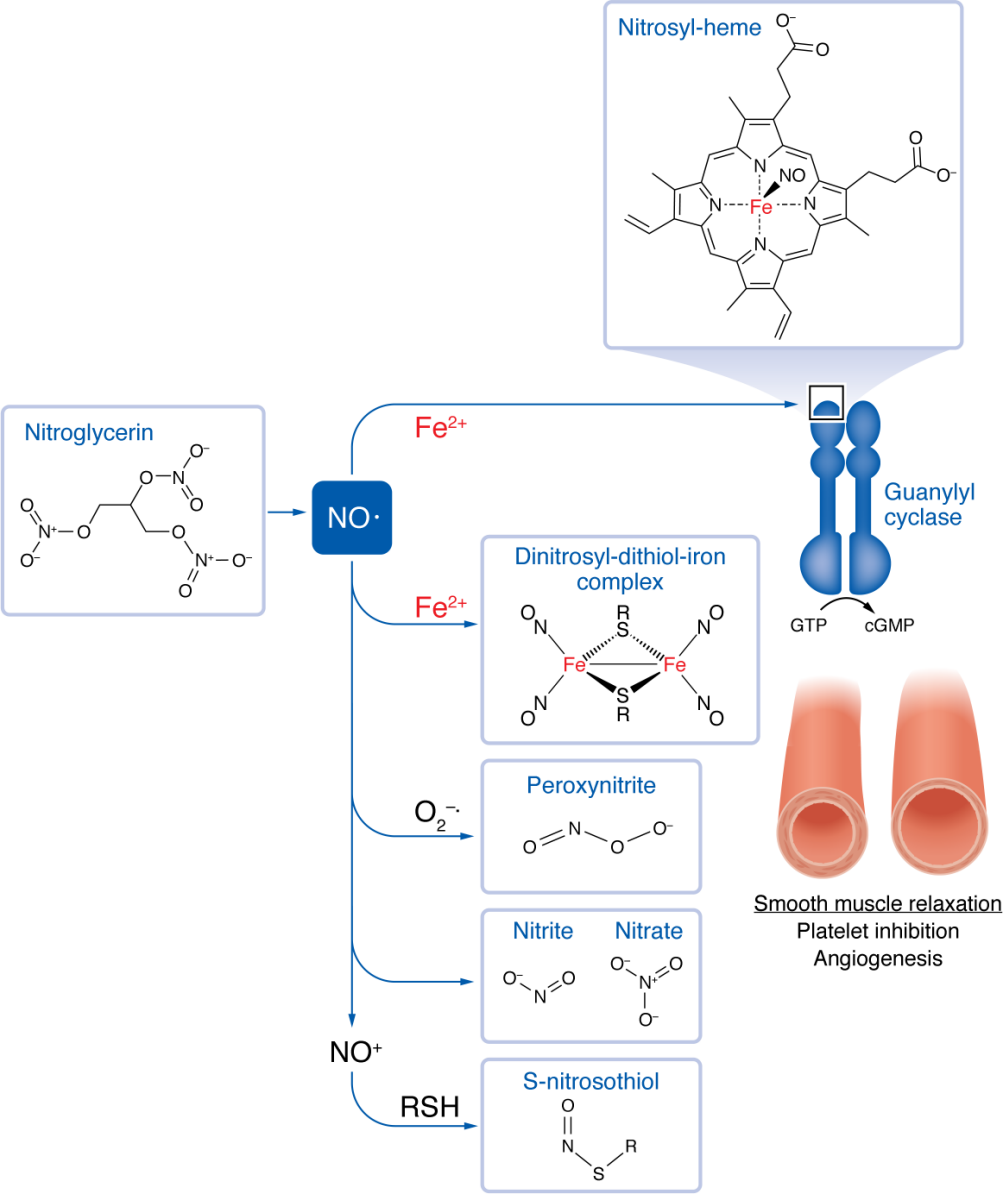
Biochemical reactions involving NO contribute to metabolic outcomes in the vasculature. The interaction between reactive NO and heme iron or other interactants provide molecular sources for many biochemical processes and functional effects in mammals. One example includes the activation of guanylyl cyclase by the nitrosyl-heme prosthetic group, resulting in vasodilation. Notably, nitroglycerin yields NO, which results in vasodilation. DNICs, generated within cells, may also act as nitrosating intermediates with involvement in vascular signaling. In combination with superoxide, NO forms peroxynitrite, which may serve as an oxidizing substrate. Other effectors of vascular function include nitrite and nitrate, which affect vasodilation, platelet function, and angiogenesis. Low-molecular-weight *S*-nitrosothiols, via NO^^+^^, also provide a source for protein modifications that promote effects such as vasodilation and platelet inhibition. RSH, sulfhydryl species.

## Version 1 02/15/2024

Electronic publication
